# Impaired Perception of Facial Motion in Autism Spectrum Disorder

**DOI:** 10.1371/journal.pone.0102173

**Published:** 2014-07-23

**Authors:** Justin O’Brien, Janine Spencer, Christine Girges, Alan Johnston, Harold Hill

**Affiliations:** 1 Centre for Research in Infant Behaviour, Department of Psychology, Brunel University, Uxbridge, United Kingdom; 2 Cognitive, Perceptual and Brain Sciences, Psychology and Language Sciences, University College London, London, United Kingdom; 3 School of Psychology, University of Wollongong, Wollongong, New South Wales, Australia; Tel Aviv University, Israel

## Abstract

Facial motion is a special type of biological motion that transmits cues for socio-emotional communication and enables the discrimination of properties such as gender and identity. We used animated average faces to examine the ability of adults with autism spectrum disorders (ASD) to perceive facial motion. Participants completed increasingly difficult tasks involving the discrimination of (1) sequences of facial motion, (2) the identity of individuals based on their facial motion and (3) the gender of individuals. Stimuli were presented in both upright and upside-down orientations to test for the difference in inversion effects often found when comparing ASD with controls in face perception. The ASD group’s performance was impaired relative to the control group in all three tasks and unlike the control group, the individuals with ASD failed to show an inversion effect. These results point to a deficit in facial biological motion processing in people with autism, which we suggest is linked to deficits in lower level motion processing we have previously reported.

## Introduction

The human body conveys an abundance of information necessary for mediating socio-emotional communication [1]. Bodily movements, facial expressions and eye gaze shifts allow us to extract information from others. We can then use this to understand their thoughts, intentions and moods [2]. Without the ability to perceive this information, social interaction would be difficult. Autism spectrum disorder (ASD) is a heterogeneous developmental disorder, characterised by a severe impairment in social communication and interaction. One factor may be due to abnormalities in the mechanisms responsible for biological motion (BM) perception.

BM has been widely studied in ASD, yet the current data is equivocal. The first discrepancy concerns what aspect of this perception is actually impaired in ASD. A number of studies report that the deficit only exists when perceiving *emotional* BM [3]–[6]. For example, one study found that high-functioning children with ASD only experienced difficulty perceiving point-light emotions. Performance was relatively normal on tasks involving body actions or inanimate movement [7]. Similarly, impairment has been observed when those with autism identify point-light bodily expressions of anger, happiness and disgust [8]. By contrast, there is evidence of the impairment extending to *non-emotional* stimuli too [9]–[11]. Blake *et al.,* asked children with autism to state whether a brief point-light animation represented a body or not. Such task was relatively unemotional, and should have evoked a normal performance. This was not the case however; those with autism still made many more errors compared to the control group [12]. These findings were later replicated by Annaz *et al.,* [13] and Nackaerts *et al.,* [14], further suggesting that all elements of BM perception is weakened in ASD.

The second discrepancy regards data which conversely report intact BM mechanisms in ASD. One study presented participants with point-light displays (walking figures, translating triangles, or translating unfamiliar shapes) embedded in noise and asked them to determine the direction of movement. Those with ASD performed similarly to controls across all three tasks [15]. Murphy and colleagues also showed that ASD participants could successfully identify the direction in which a point-light walker (embedded in noise) was moving in. The authors suggest that ASD participants were able to integrate local motion cues to produce a coherent perception of BM [16].

Such findings may reflect an experimental bias caused by testing different age groups. Studies conducted with ASD children consistently report a deficit in BM perception [7], [12], [17], whereas the adult data is less conclusive. Perhaps such perception improves with chronological age [4], [13]. It is possible that older ASD subjects acquire compensatory mechanisms, and thus perform similarly to typically developing subjects on BM tasks. For example, the absence of global processing may force ASD adults to acquire superior processing with local cues [18]. Alternatively, factors such as symptom severity [12] or general intelligence [19] could affect the ability of ASD participants to perceive BM. Indeed, Rutherford and Troje [20] showed that only those ASD individuals with a low IQ had a poor perception of BM.

While the existing data has been highly informative, there is a paucity of research exploring the perception of facial BM. Yet, the face facilitates social interaction by providing both categorical (identity, gender, age) and qualitative (emotions, intentions, thoughts) visual information. If we are to assume that BM deficits are accountable for impairments in social cognition, then it is essential we actually investigate this using facial BM. To our current knowledge, only two research groups have utilized dynamic face stimuli when examining emotion recognition in ASD. Pelphrey *et al.,* [21] and Sato *et al.,* [22] observed functional abnormalities within a number of areas responsible for social perception (e.g., amygdala, fusiform gyrus and superior temporal sulcus) and visual processing, respectively. This constellation of neural abnormalities may therefore cause impairments in facial motion perception [23].

Facial BM perception in ASD is thus beginning to attract the attention of researchers. Much of this cognition however is still left relatively unexplored. We therefore utilized averaged facial motion captures [24] to investigate whether ASD individuals could use facial motion to discriminate between sequences, identify different unfamiliar individuals and categorise genders. Previous studies with typically developing subjects have shown that facial motion cues are sufficient in aiding these discriminations [24]–[29], but see others for conflicting data [30], [31]. It demonstrates that 3D dynamic information provides a better structural depiction of the face, perhaps by increasing and refining view-points or conveying idiosyncratic mannerisms typical to the individual [27], [32], [33].

Presentations of facial motion varied between upright and inverted stimuli. This allowed us to observe significant differences between the ability of ASD and control participants in their perception of facial motion. Studies conducted with static faces have demonstrated that inverted stimuli affect face recognition by disrupting configural processing and early structural encoding [34], [35]. As a result, accuracy on such face perception tasks is significantly reduced. Previous research has not found this effect in ASD [36], [37], [38], suggesting that subjects fail to utilize configural strategies and instead rely on feature-based processing [39]. Indeed, children with autism have a superior perception of individual facial features and are better at recognising partially obscured faces than controls [40]. Recent reviews however have highlighted inconsistencies surrounding this manipulation in ASD [41]. This may be a consequence of studies implicating the often unrealistic static facial display.

Thus, in the current study we sought to address two questions: (1) are ASD subjects able to perceive facial motion, and use such information when making judgments about sequence, identity or gender and; (2) is the performance of ASD subjects unaffected by inversion paradigms, therefore confirming feature-based processing of faces in ASD. Answering such questions might shed light on whether an impaired perception of facial BM contributes to the social cognitive impairments seen in this disorder.

## Method

### Ethics Statement

Full ethical approval was obtained from the Brunel University Social Science ethics committee. All participants gave written informed consent prior to the study and received a debriefing document following their participation.

### Participants

Adults with ASD were recruited from residential care facilities in the London area for individuals on the autism spectrum. Fourteen high-functioning individuals with ASD (*subtype = Asperger’s Syndrome, DSM-IV-TR code: 299.80*) took part (11 male, 3 female, *mean age = *33. 85 years, *range* = 22–51 years, *Autistic Quotient (AQ) score* = 26.40). All fourteen participants were diagnosed by psychiatrists specializing in developmental disorders. The diagnoses were based on the DSM-IV-TR (APA, 2000) criteria. Eleven participants were right handed, and three were left handed. The highest level of education varied within the ASD group from high school to the first year of a university degree. Fourteen individuals with typical development also took part (7 male, 7 female, *mean age* = 31.14 years, *range = *21–56 years, *AQ score* = 14.45), and were recruited from Brunel University (London, UK). All participants were right handed and the highest level of education ranged from high school to university degrees (or the equivalent). None of the ASD or control sample had any history of other neurological or psychological conditions and reported normal or corrected-to-normal vision.

Groups were matched on age and scales of non-verbal analytic intelligence (Standard Progressive Matrices) [42]. We used such measures of IQ as ASD participants had already received standard IQ testing within a year prior to the current study. It was therefore necessary to use other measures to avoid practise effects. Both groups of participants were also tested on their ability to perceive static faces [43], which they all completed within the normal range. Any difficulty then experienced during experimental testing would therefore suggest a specific problem in facial motion perception, rather than a generalised impairment in face processing. Characteristics of control and ASD participants are presented in Table 1.

**Table 1:** Characteristics of adults with ASD and the control group.

Variable	Controls	ASD	*P-value*
***n***	14	14	–
**Age, years**			
* * ***Mean***	31.14	33.85	0.570
* * ***Range***	21–56	22–51	–
**SPM** *			
* * ***Mean***	49.00	42.31	0.070
* * ***Range***	31–56	19–52	–
* * ***% score***	82	71	–
**Benton Facial Recognition** *			
* * ***Mean***	47.79	45.79	0.287
* * ***Range***	45–54	36–52	–
* * ***% score***	89	85	–
**AQ** *			
* * ***Mean***	14.45	26.40	0.001
* * ***Range***	12–20	21–36	–

*Maximum possible scores for the Standard Progressive Matrices (SPM) = 60; for the Benton Facial Recognition test = 54; scores between 11 and 22 on the Autistic Quotient (AQ) scale were considered average.

### Stimuli

Facial animations were generated by applying the motion captured from 12 actors to a three-dimensional averaged face (see [24]). Each animation was of an ‘actor’ telling simple question and answer jokes. These jokes elicited natural facial expressions and movements. The animations were all identical and only differed in the way they moved. This allowed the experimenters to measure biological motion independently, without information from audio or other visual cues influencing the responses. An inverted version of each stimulus was generated in Matlab by manipulation and re-encoding of the original stimulus video file. The present study used these stimuli to investigate whether individuals with ASD could perceive and discriminate facial motion.

### Procedure

The dynamic face stimuli were presented using an LCD display with resolution 1024×768 and 60 Hz refresh rate. Viewing distance was approximately 60 cm, at which distance the 30 cm×22.5 cm display subtended an angle of 26.6°×20.6°. The height of the average face was approximately 10.5°, and the frame rate of the animation was 30 FPS. Instructions were given verbally and the experimenter recorded participants’ verbal responses manually. Each participant took part in all of the experimental conditions.

There were three experimental tasks (sequence, identity and gender discrimination), each with two manipulations (upright and inverted). Each condition had 21 trials, plus 8 attention control trials. The first condition was the *sequence discrimination task.* Participants viewed a single facial animation displayed in the centre of the screen. They then viewed the same animation again, plus a completely different animation, shown side-by-side on the screen. All animations were presented for 5 seconds. Using a two-alternative forced choice procedure, participants had to indicate which stimuli (left or right on the screen) were present in both presentations. A similar format was used for the *identity discrimination task.* A single facial animation was presented, followed by another two animations. One of the test animations was from the same actor telling a different joke (the correct response), and the other was of a second actor telling another different joke. The *gender discrimination test* required participants to view a single animation, and respond whether it was male or female. Conditions were randomised to avoid familiarity effects. Please see supporting information for example PowerPoint files demonstrating the procedure for each task (PowerPoint S1–S3).

All conditions included attention-control trials. On every fourth trial, the correct responses were indicated with a blue arrow placed above the animation. The arrow was present at the beginning of the trial, and remained on the screen until the participant made their response. Participants were aware that the arrow indicated the correct answer. The responses to these trials were not included in any subsequent analysis. The purpose of these trials was to be able to eliminate from consideration any data provided by a participant in a given condition who did not show full attention to the task. All control and ASD subjects completed the attention control trials without error. Consequently no data was discarded. It is common practice in the psychological literature of developmental disorders to match participants in experimental and control groups by chronological age and verbal mental age. Such measures are insufficient to ensure that the only difference in responses from participants is due to the perceptual factors under investigation. Observer responses can be influenced by fatigue, boredom or intermittent confusion, for example, and the use of attention-control trials can be used as a conservative criterion for rejecting any data where there is a possibility of non-perceptual factors influencing responses [44].

## Results

**Figure 1:**
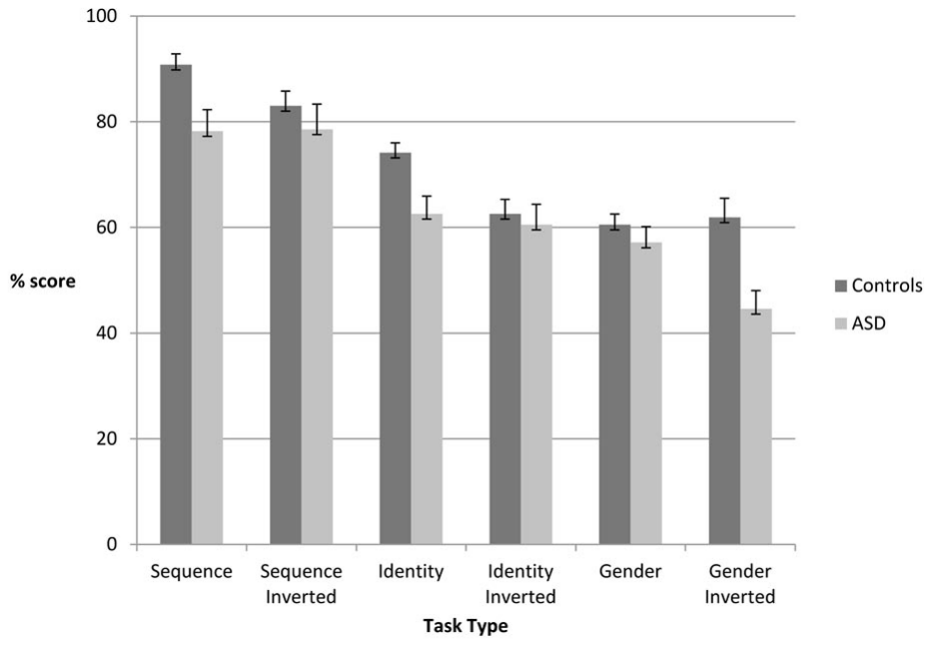
Proportion of correct responses on each task for the control and ASD subjects.

Figure 1 shows the proportion of correct responses made by ASD and control subjects for each task. One-sampled t-tests were used to compare the performance in each condition with the chance response rate of 10.5 (50%). For the ASD group, performance was not significantly above chance level of 0.05 (Bonferroni corrected) in the (1) inverted identity discrimination; (2) upright gender discrimination; and (3) inverted gender discrimination tasks. The control group did not perform above chance on the inverted gender discrimination task.

A mixed design ANOVA indicated a significant three-way interaction between *task type* (sequence, identity, gender) *orientation* (upright, inverted) and *group* (ASD, controls) (*F*_(2, 52)_ = 9.965, *p*<.001). We observed a further significant interaction between *orientation* and *group* on the facial motion sequence (*F*_(1, 26)_ = 5.236, *p* = .030), identity (*F*_(1, 26)_ = 4.826, *p* = .037), and gender (*F*_(1, 26)_ = 9.071, *p* = .006) discrimination tasks.

A follow up one-way ANOVA revealed a significant difference between the performance of the ASD and control group on both the *upright sequence* (*F*_(1, 26)_ = 7.73, *p* = 0.01) and *upright identity F*_(1, 26)_ = 9.16, *p*<0.01) discrimination tasks. Compared to control participants, the ASD sample made more errors during these tasks. Further, there were no significant differences between ASD and control participants on the *upright gender* discrimination task (*p*>0.05). However, the difficulty of this task was such that performance was above chance only for the control group in the upright condition.

The same analysis was applied to data from inverted conditions (Table 2). There were no significant differences between the ASD and control group for *inverted sequence* (*F*_(1, 26)_ = 0.90, *p*>0.05) and *inverted identity* discrimination tasks (*F*_(1, 26)_ = 0.19, *p*>0.05). Inverted facial motion affected the controls, decreasing their correct response rate. For those with ASD, there was no difference in performance on upright and inverted conditions. Inverting the stimuli during *gender discrimination* tasks did however produce a significant difference between the control and ASD group (*F*_(1, 27)_ = 11.89, *p* = 0.002). This finding may be discounted though by the below chance performance evident in both groups.

**Table 2:** Mean scores (Standard Deviations) and results from a one-way ANOVA.

Variable	Mean (SD)		Differences between groups (One-way ANOVA)		
	*ASD*	*Controls*	*F*	*df*	*P-value*
**Sequence**	16.43 (3.18)	19.07 (1.59)	7.73	1, 26	0.010
**Sequence Inverted**	16.50 (2.93)	17.43 (2.21)	0.90	1, 26	0.352
**Identity**	13.14 (2.63)	15.57 (1.45)	9.16	1, 26	0.006
**Identity Inverted**	12.71* (3.05)	13.14 (2.14)	0.19	1, 26	0.671
**Gender**	12.00* (2.35)	12.71 (1.59)	0.89	1, 26	0.355
**Gender Inverted**	9.36* (2.74)	13.00* (2.68)	11.89	1, 26	0.002

*Indicates any result not above chance.

## Discussion

Impairments in perceiving BM has been suggested to underlie the social cognitive deficits in ASD [30], [31]. We extended such investigations to facial motion perception, examining whether ASD subjects could use these cues to make certain categorizations. Participants therefore engaged in sequence, identity and gender discrimination tasks.

Our findings indicate that although the current ASD sample were able to recognise static faces from the Benton’s test, they were still poor on tasks requiring them to discriminate between upright sequences of facial motion. They were also unable to use upright facial motion as a cue for identity. An inability to recognise a number of different individuals from basic motion patterns may significantly impact social cognition in ASD. Moreover, unlike the control group, ASD subjects did not show an inversion effect in either task even though facial motion appears to be processed by a system tuned to upright faces [24]. It would appear then that the neural mechanisms responsible for facial motion perception are weakened in ASD. This finding is comparable to other investigations which have utilized point-light body motion stimuli [4], [7], [8], [10], [12], [14].

We are disinclined to suggest poor attentional abilities are at the root of the problem as all participants scored correctly on the attention-control trials in each experimental condition. Incompetent cognitive skills can also be dismissed; all participants passed the Standard Progressive Matrices test within the typical range and understood the tasks well. Perhaps the impairment in facial BM perception arises from problems in configural processing [12]. Individuals with ASD may focus heavily on a particular and perhaps trivial feature, at the expense of global motion [45]. There is some evidence to support this view. Van Boxtel and Lu [18] measured accuracy on a central counting paradigm while task-irrelevant biological movements were presented in the periphery of participants with low and high autistic traits. Stimuli were either intact or spatially scrambled. Subjects with fewer autistic traits were found to involuntarily process global aspects of BM even when it negatively affected their central task performance. Individuals with high autistic traits however did not show this attentional distraction, performing identically on the central task in the scrambled and intact conditions. An absence in configural (or global) processing would certainly support the indifference to orientation present in our current ASD sample. Engaging more in featural or local processing would by-pass the disruption caused by inverted motion [38], [40].

The impairment could also lie within low-level visual mechanisms, specifically in the transmission of information from primary visual areas to substrates involved in social cognition [46]. This may explain why the superior temporal sulcus -a structure known for its involvement in BM processing- is often hypo-activated in ASD individuals compared to typical controls [47]–[50]. It is not unreasonable to suggest a deficit in integrating or transmitting complex perceptual information, rather than a dysfunction of a specialised social structure *per se.* Studies of motion perception in ASD have shown the etiology of such deficit to lie within weakened integration mechanisms and/or faulty visual (dorsal) pathways [51]–[55]. Abnormal connectivity between key substrates could also lead to deficits in the perception of biological movements. For example, Sato *et al.,* [22] observed decreases in the bi-directional connectivity between the primary visual cortex, middle temporal gyrus and the inferior frontal gyrus when ASD subjects viewed dynamic displays of facial emotion. Such neural impairments may therefore underlie the facial motion deficits seen in the current study.

Further, control and ASD participants did not differ in their performance on *upright gender* discrimination tasks. This result is discussed in reference to the stimuli set. Some of the facial motion captures appeared to be somewhat impassive or expressionless. As Berry [26] states, female faces are typically more animated during interaction than are male faces. For the control group, a higher percentage of animations may have therefore been incorrectly judged as male. This larger proportion of incorrect answers would then be more comparable with ASD participants, who seemed to completely guess answers as indicated by a below chance performance. The ASD participants also showed an inversion effect during this task. We are uncertain that this is a genuine effect though due to their below chance performance here.

It is possible that we are running into a floor effect on gender discrimination tasks. However, a similar study which looked at discriminating genders from facial motion found that healthy controls could only do this for 68% of the trials [26]. Such result is comparable to the 61% found in the current study. Hill and Johnston [24] also report a just above chance performance on gender discrimination tasks in typically developing individuals. These findings demonstrate that perhaps accurate gender identification relies on the presence of both facial motion and characteristic structural form cues. As the current study did not include stimuli which differed in appearance, we cannot comment on this but encourage future investigations to clarify this issue.

## Conclusion

Our data indicates that those with ASD have an impaired perception of facial motion, and are unable to use such cues when making categorical discriminations. This result is discussed in terms of faulty configural mechanisms and/or a dysfunction within visual pathways leading to key BM processing substrates. Such impairment could then contribute to the socio-emotional impairments seen within this disorder. Future studies may wish to elaborate on these results by correlating symptom severity with facial motion perception or examining abnormalities within such networks during facial motion categorisations.

## Supporting Information

PowerPoint S1 **Example of the sequence discrimination trials.**
(PPTX)

PowerPoint S2 **Example of the identity discrimination trials.**
(PPTX)

PowerPoint S3 **Example of the gender discrimination trials.**
(PPTX)
