# Supplementary material for: Impaired Perception of Facial Motion in Autism Spectrum Disorder
Source: PLoS One. 2014 Jul 23;9(7):e102173. doi: 10.1371/journal.pone.0102173 (PMC4108352; doi:10.1371/journal.pone.0102173)
Supplement: PowerPoint S2 — Example of the identity discrimination trials. (PPTX) [file pone.0102173.s002.pptx]

## Slide 1
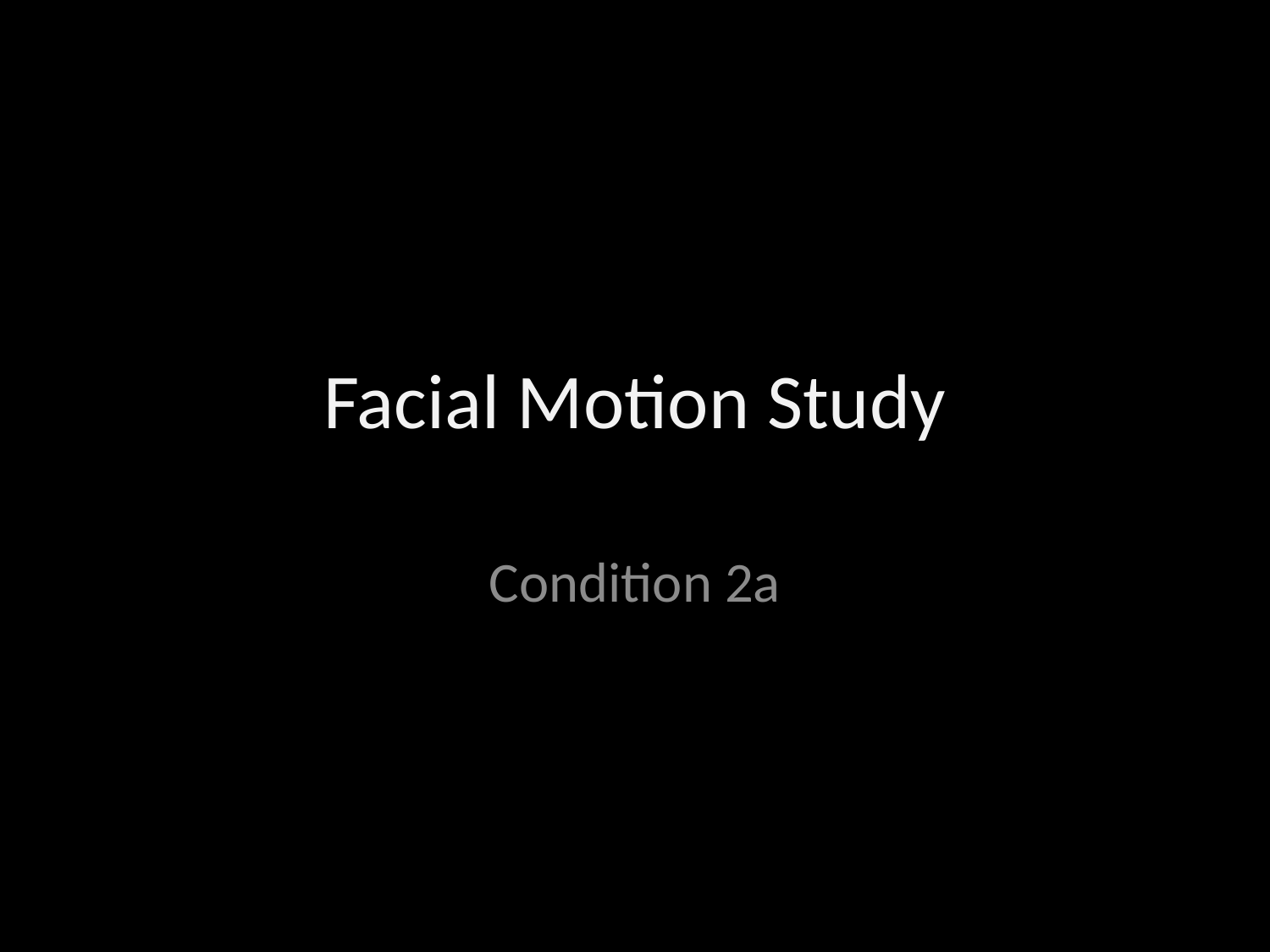

# Facial Motion Study
Condition 2a

## Slide 2
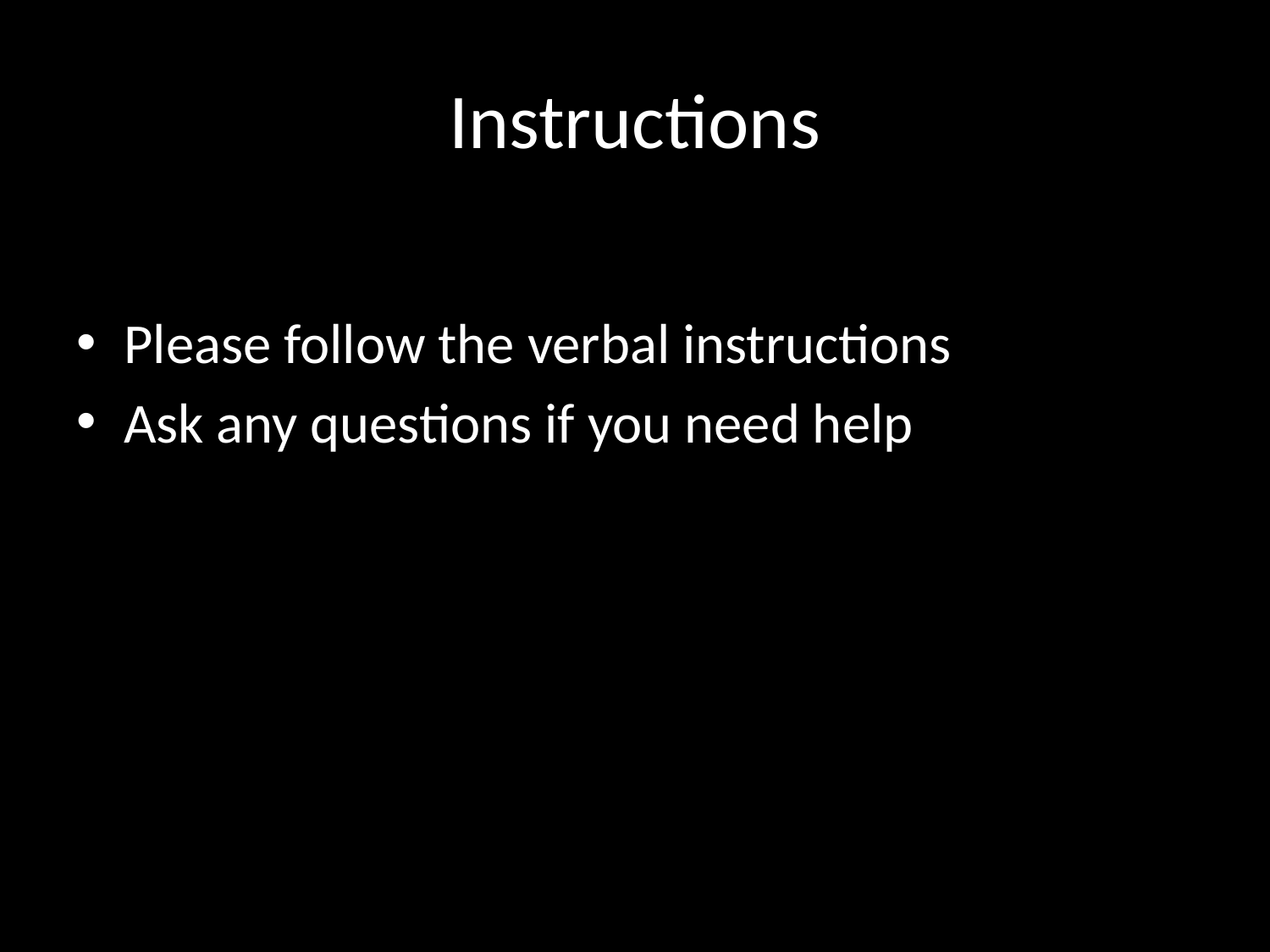

# Instructions
Please follow the verbal instructions
Ask any questions if you need help

## Slide 3
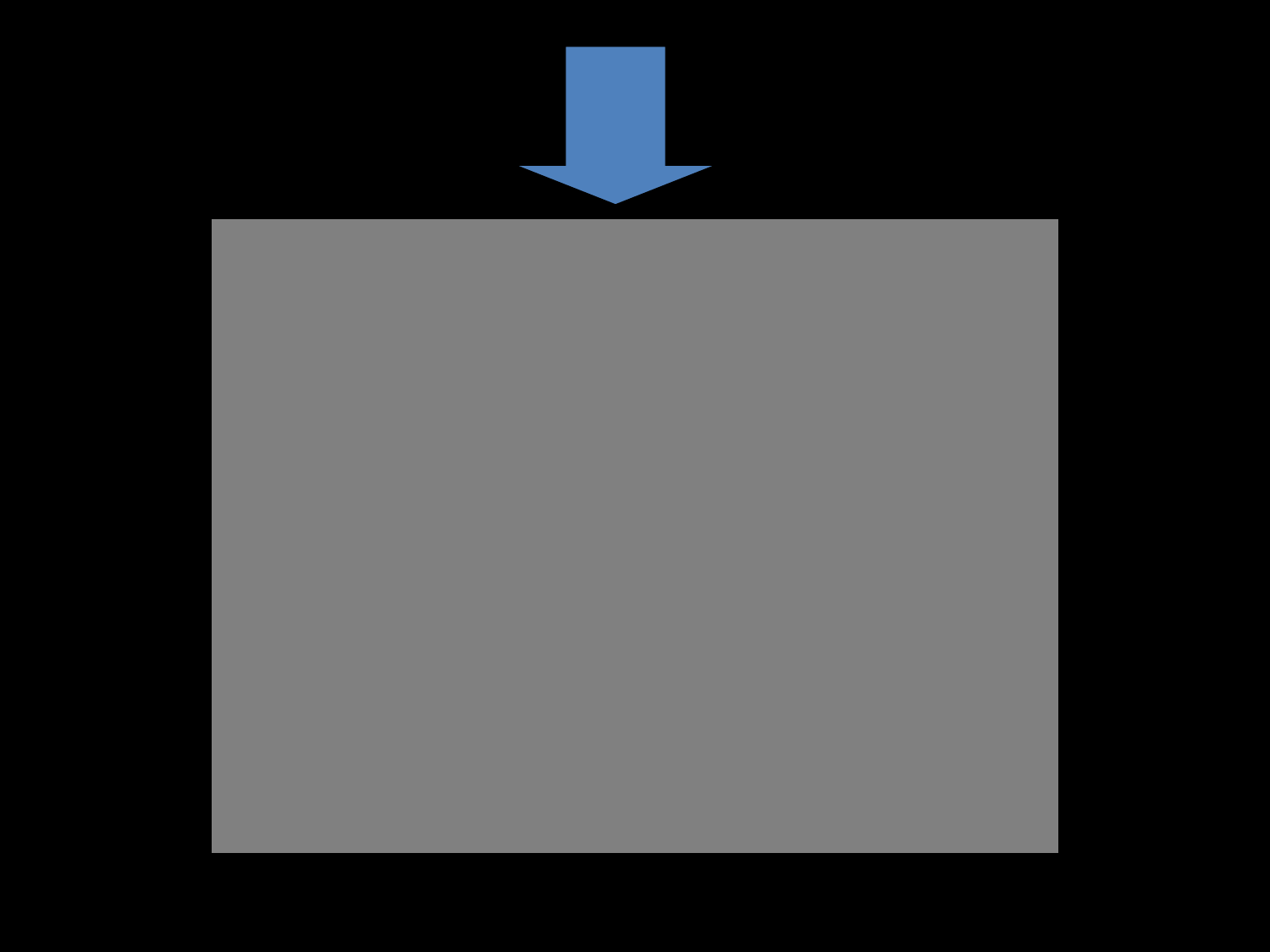

#

## Slide 4
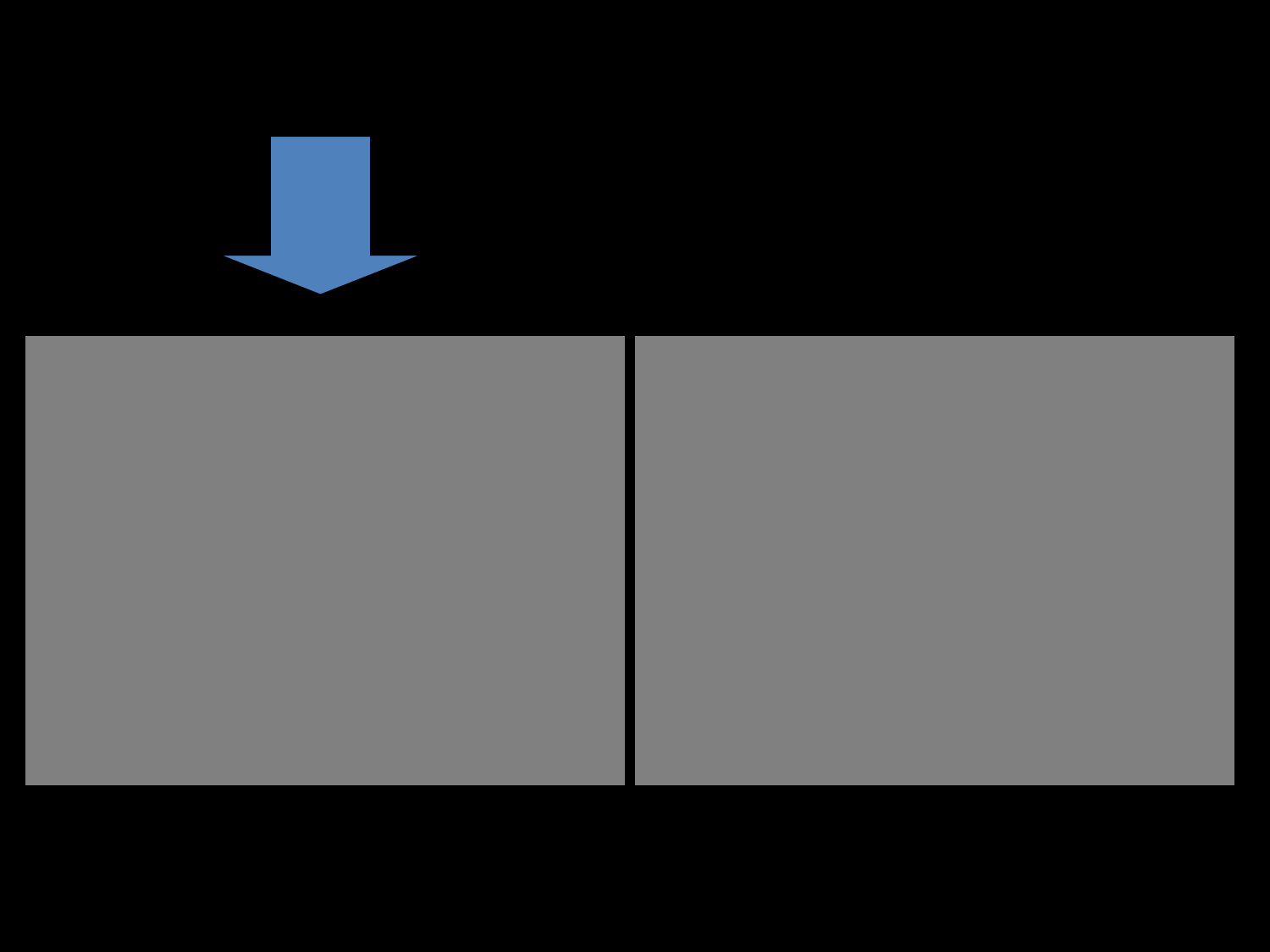

#

## Slide 5
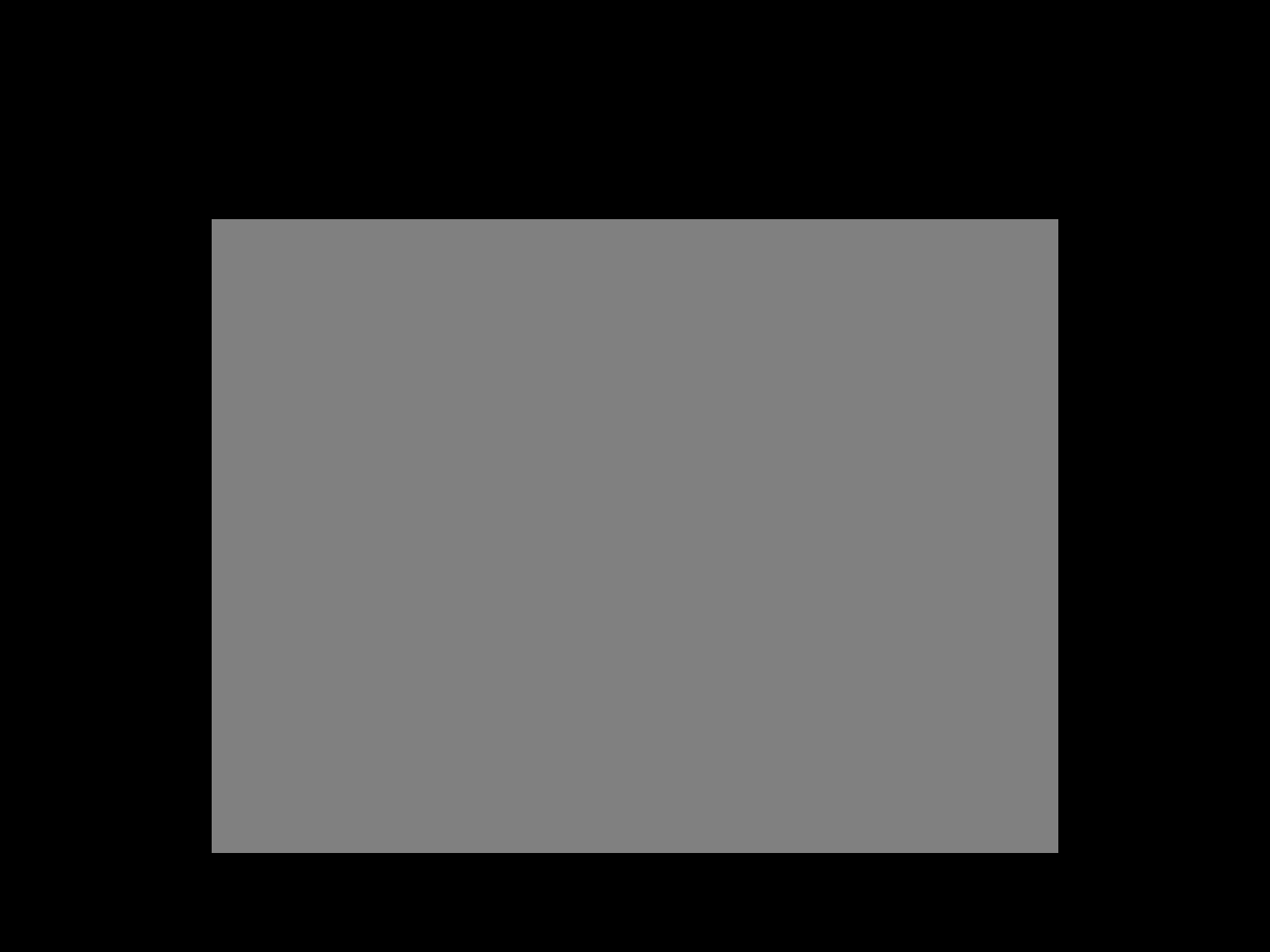

#

## Slide 6
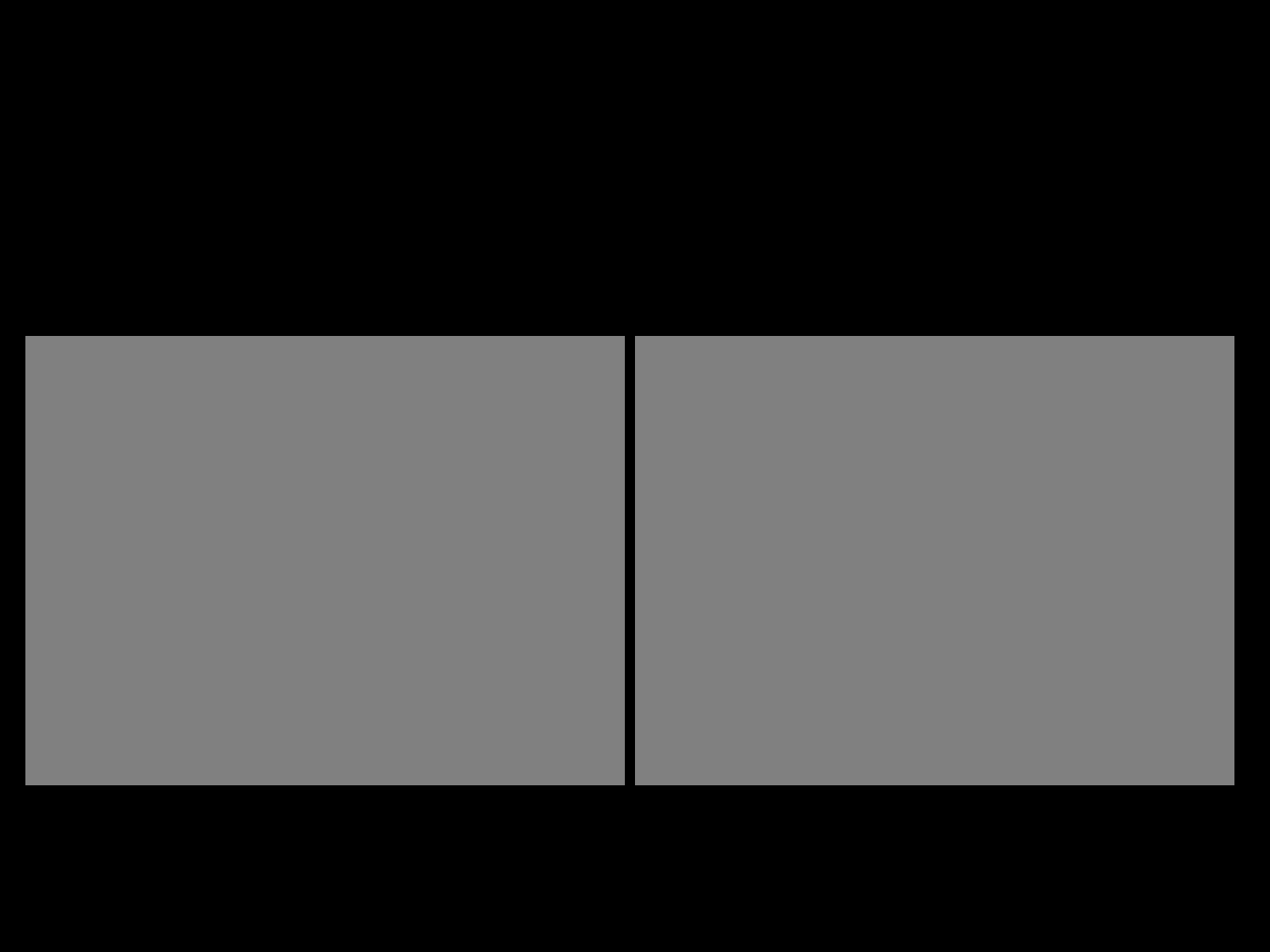

#

## Slide 7
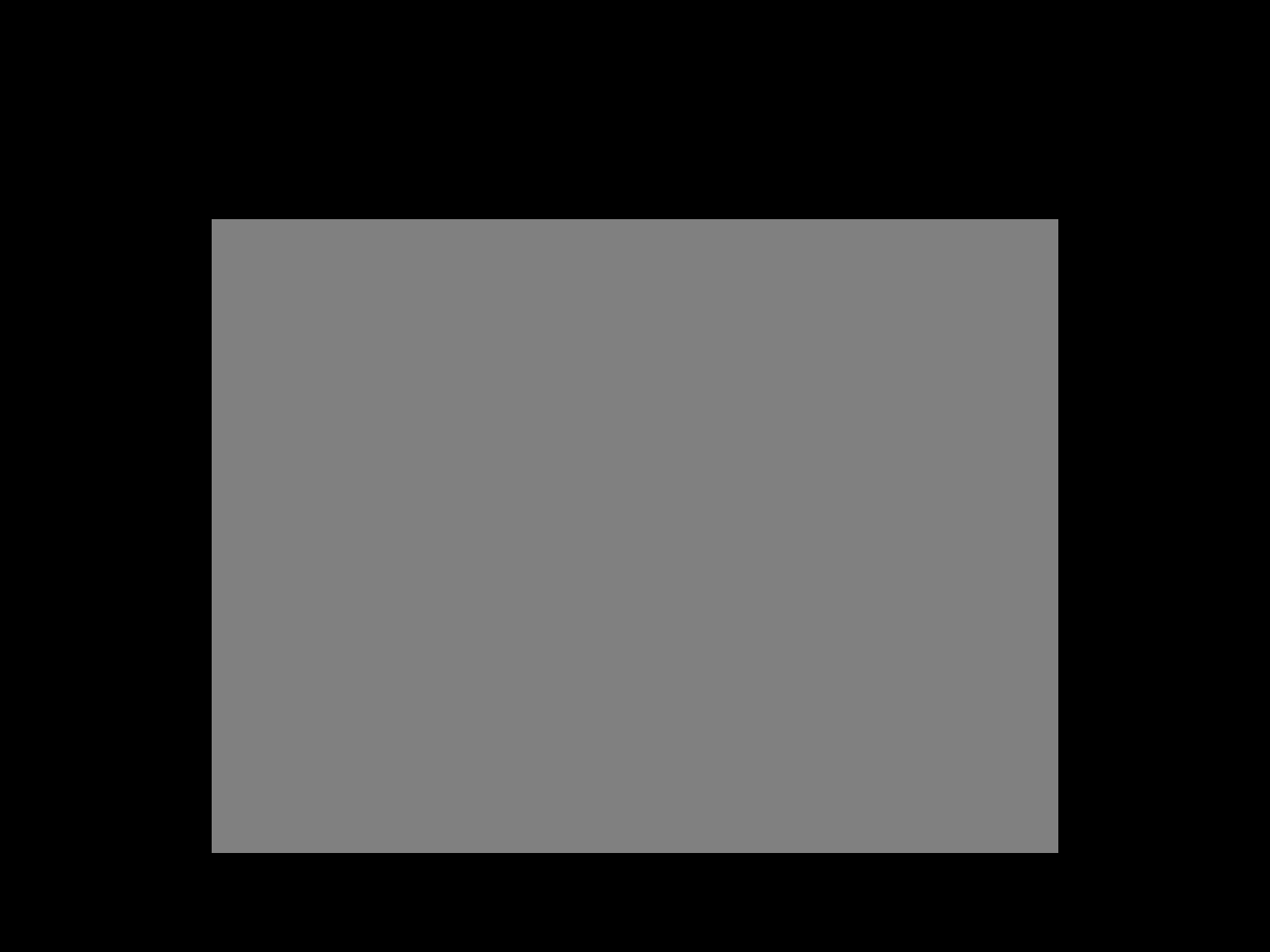

#

## Slide 8
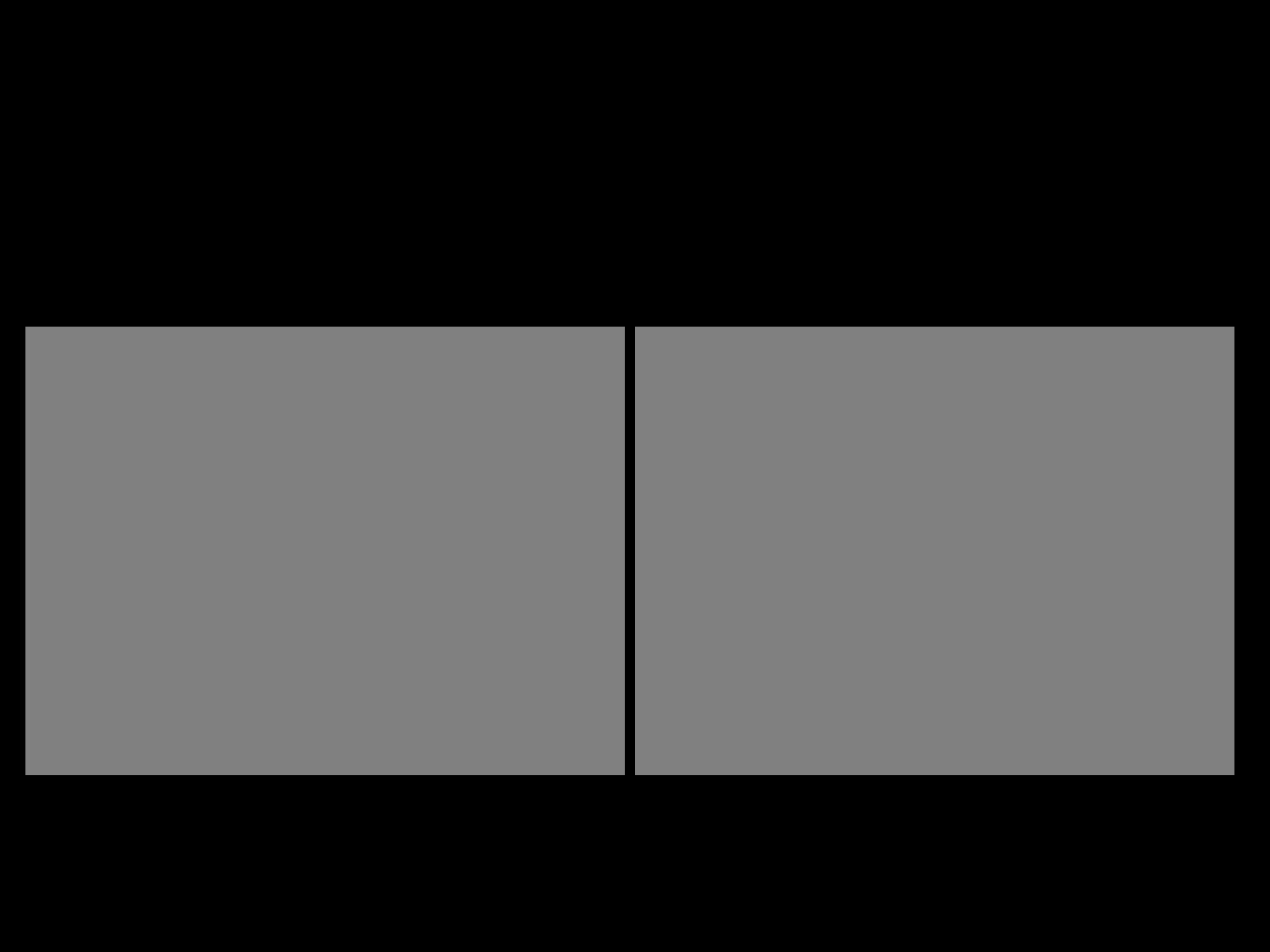

#

## Slide 9
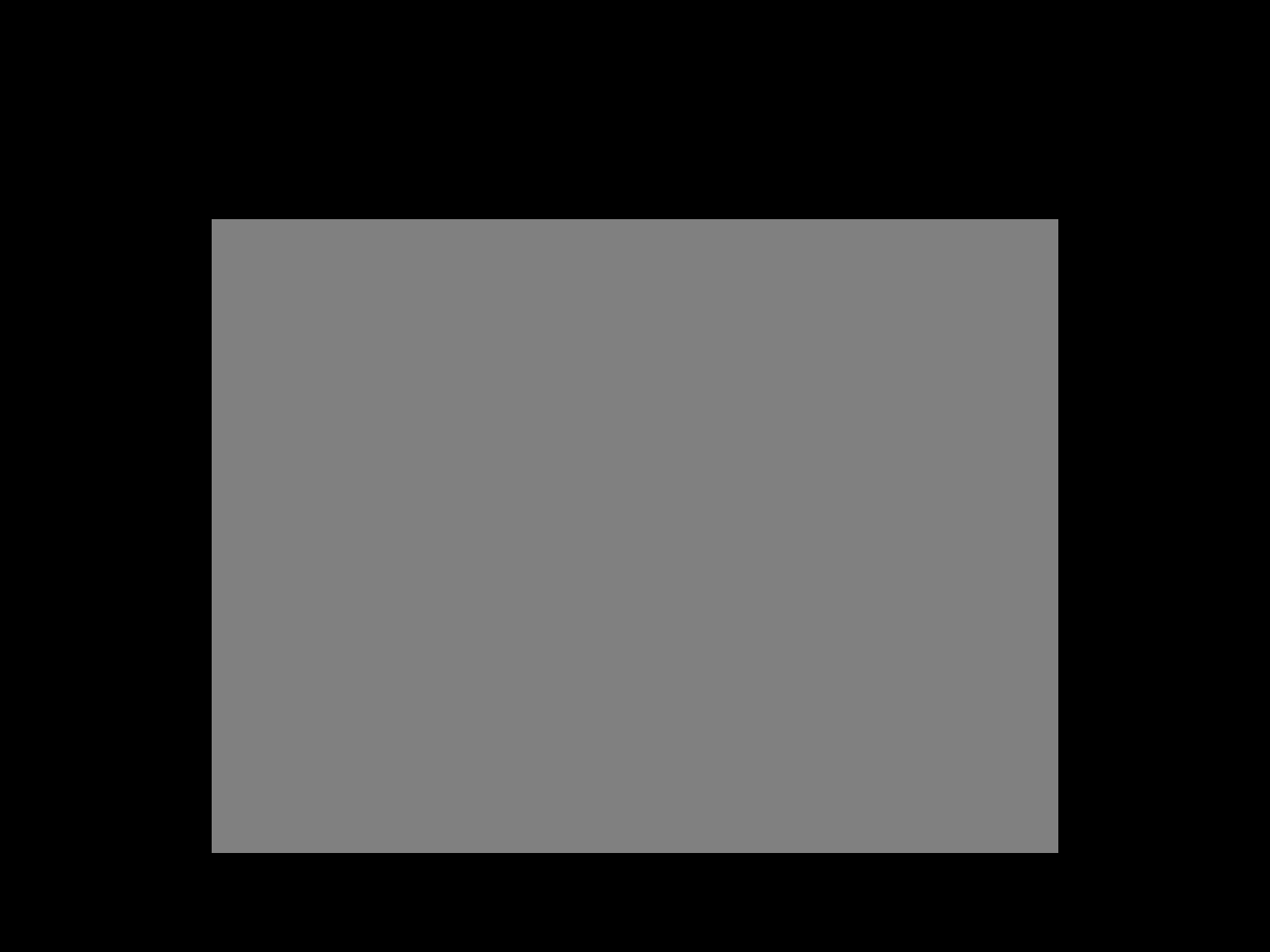

#

## Slide 10
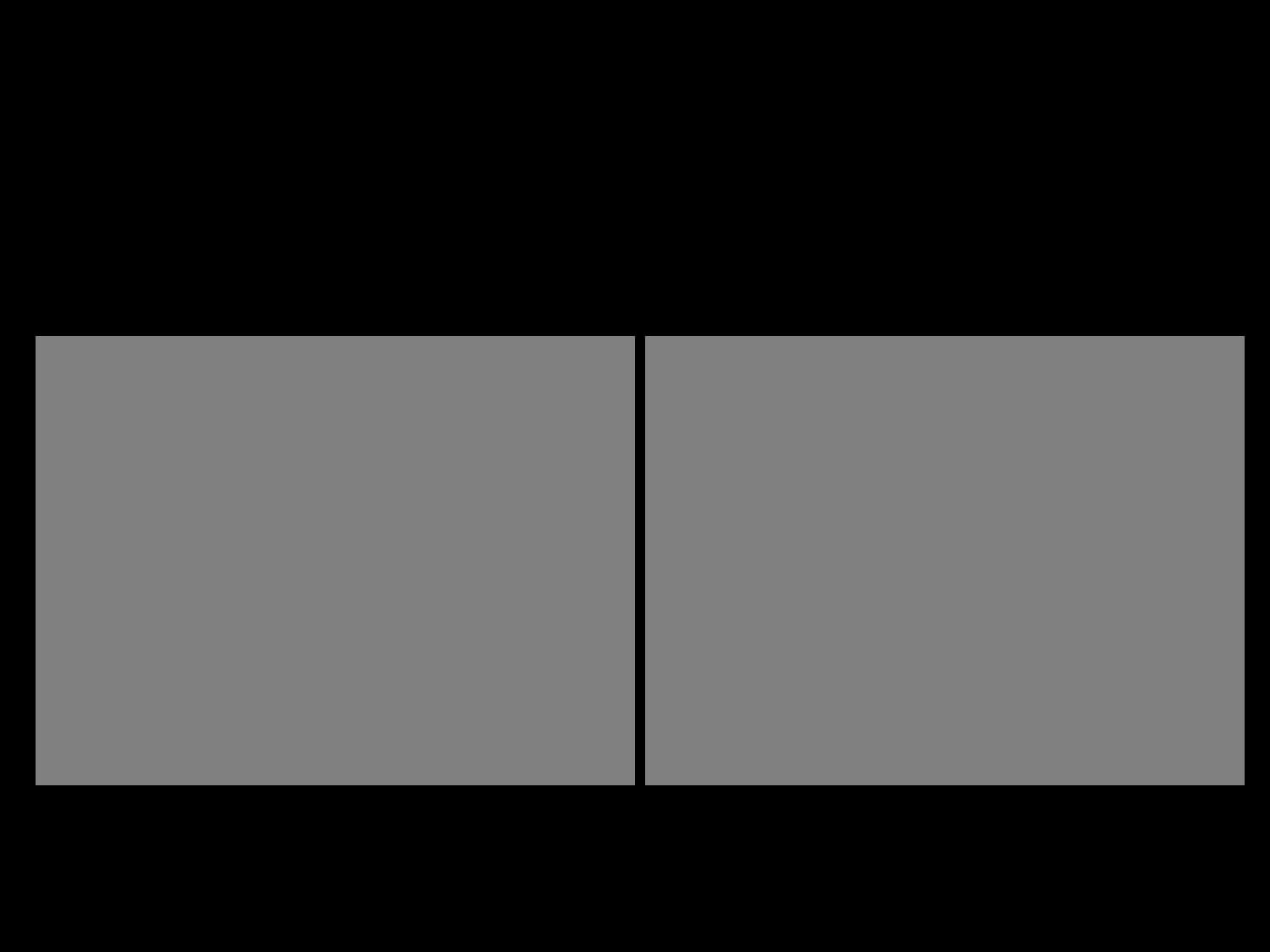

#

## Slide 11
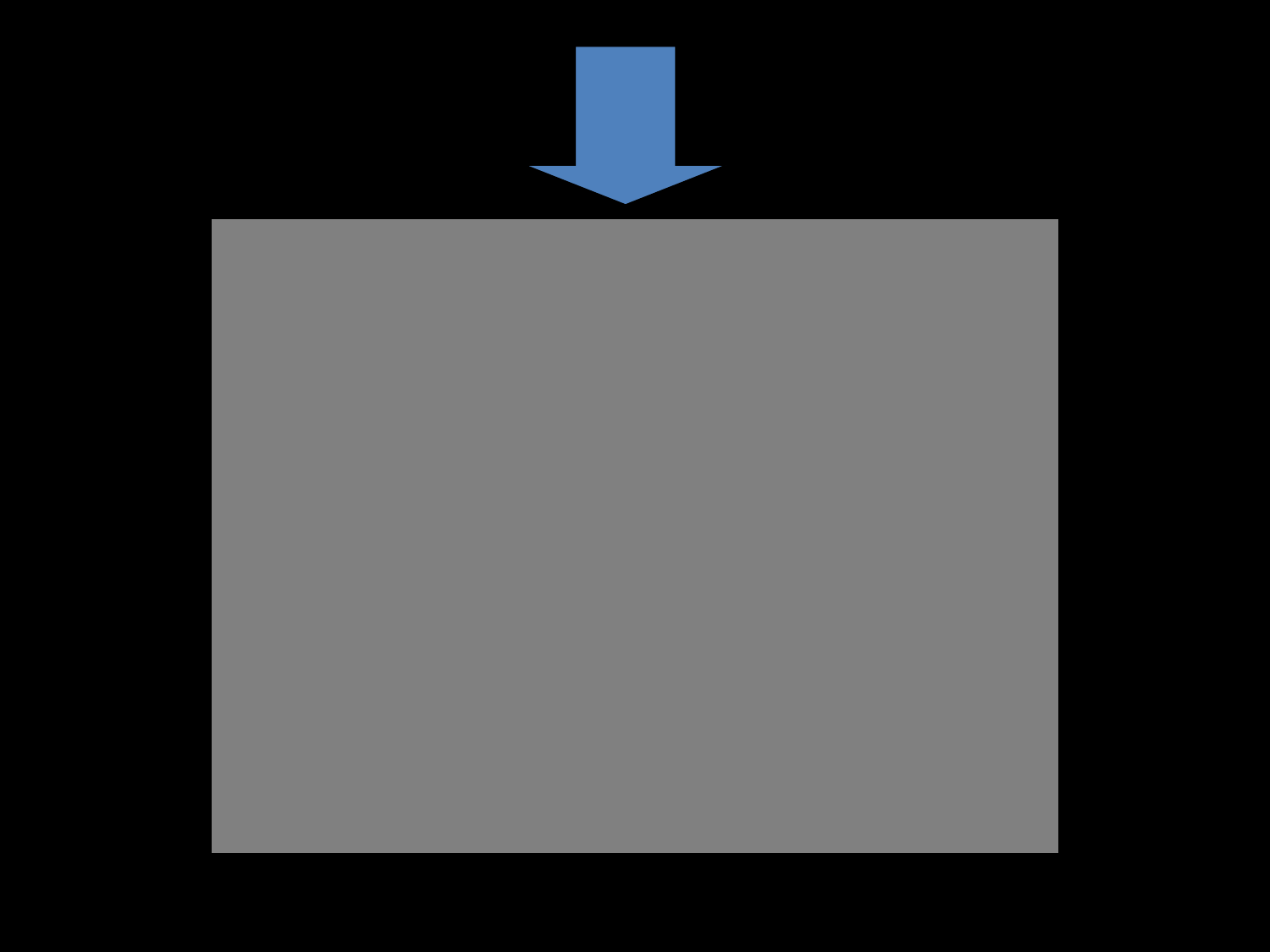

#

## Slide 12
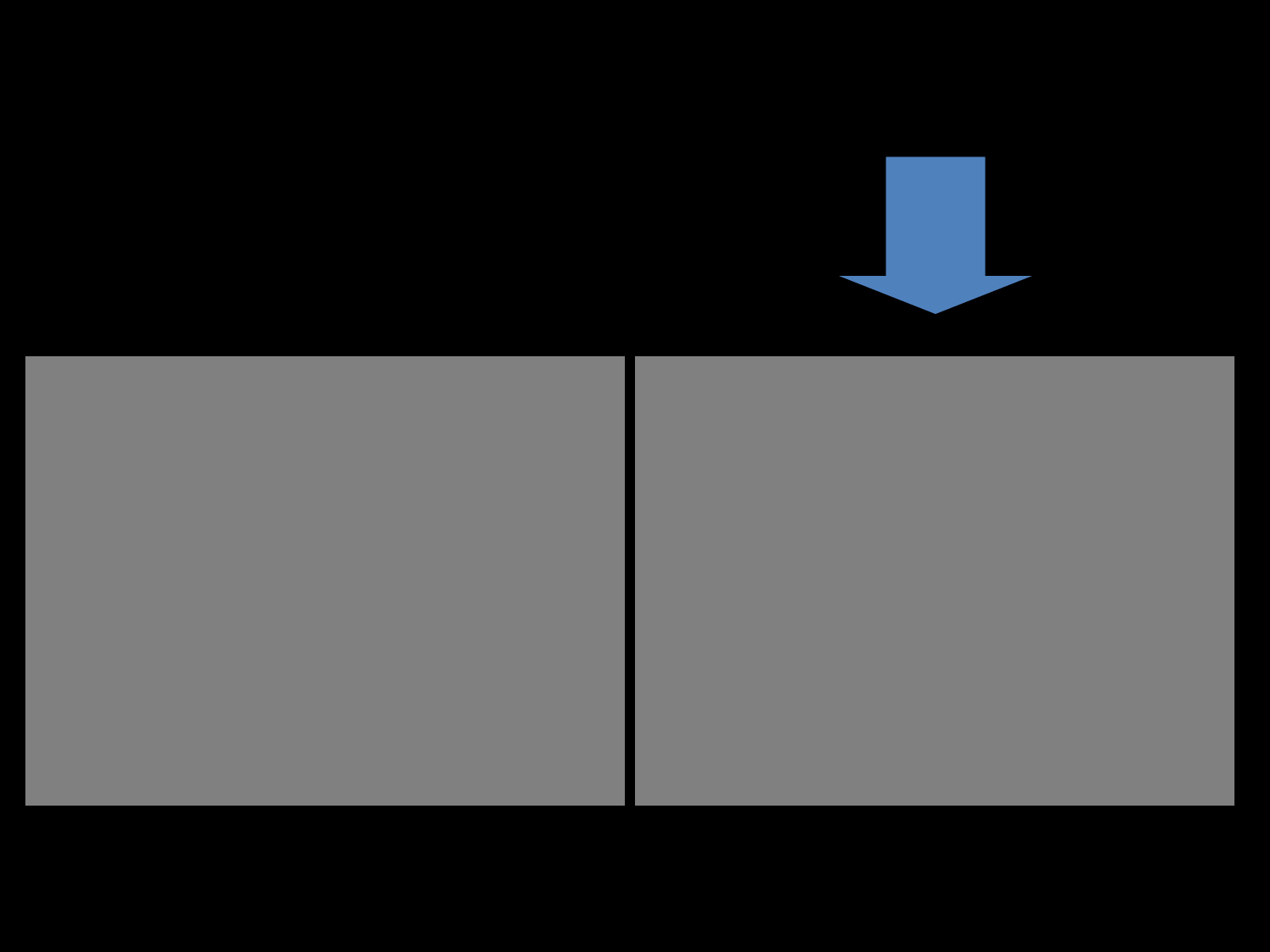

#

## Slide 13
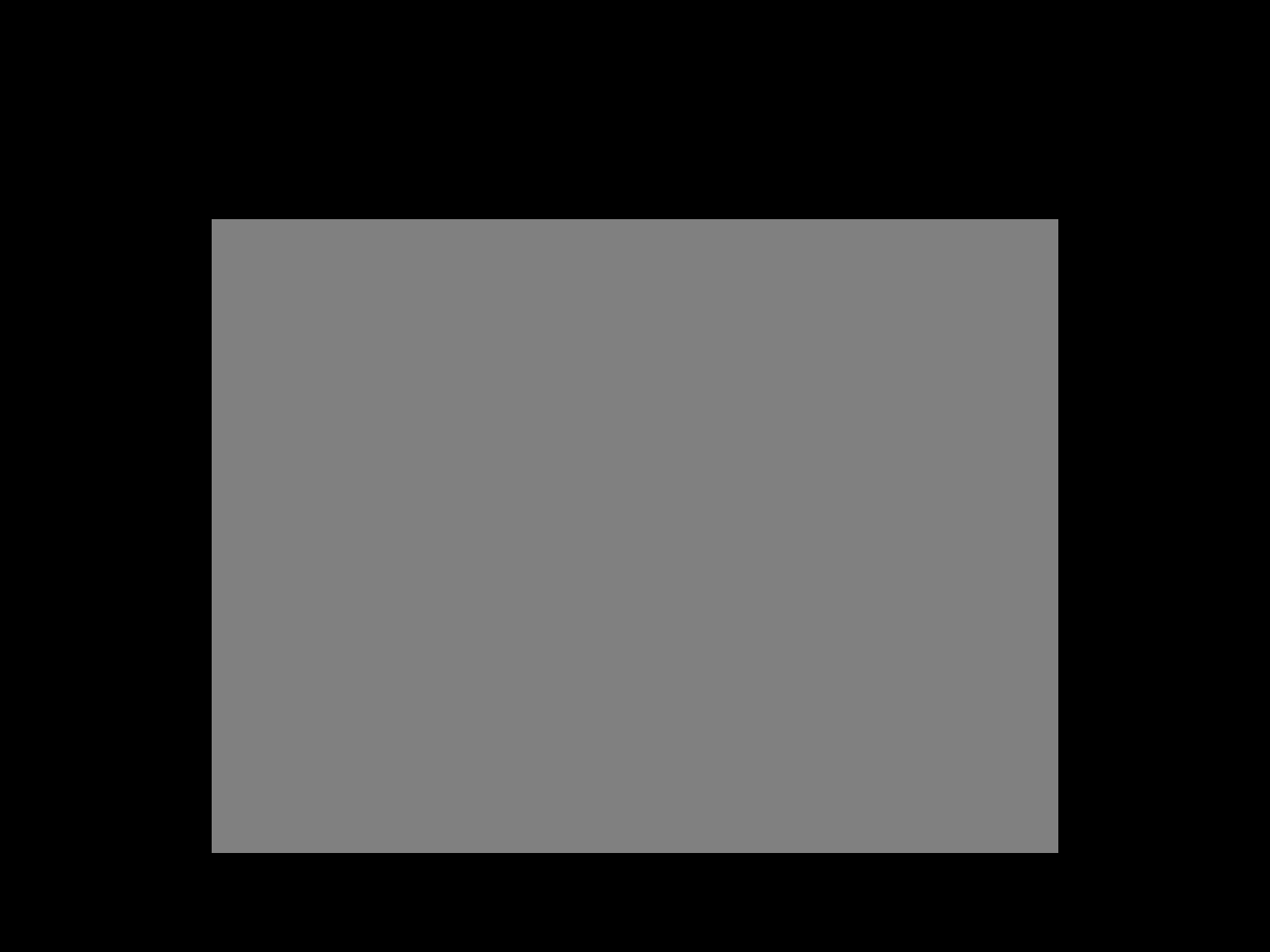

#

## Slide 14
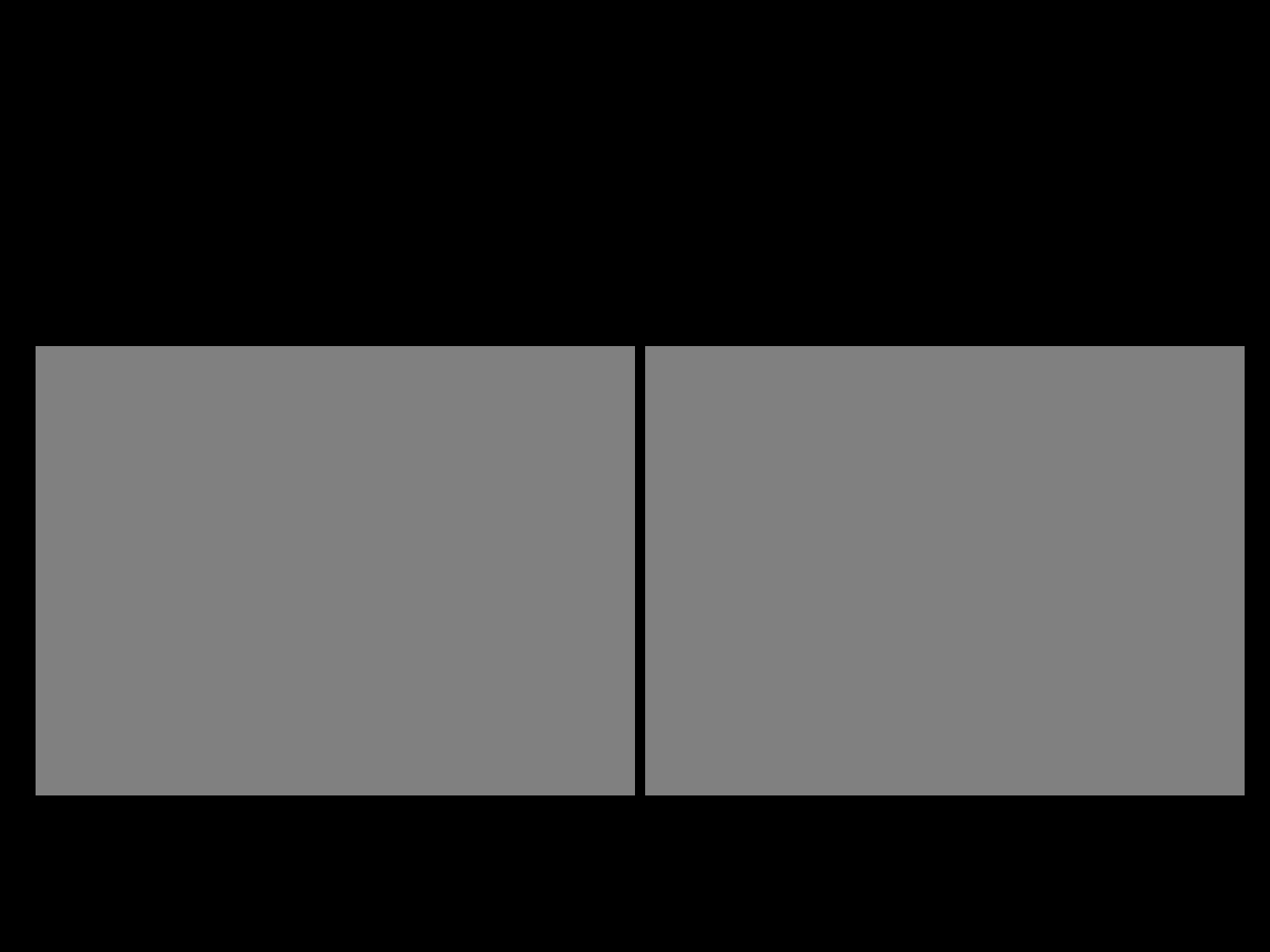

#

## Slide 15
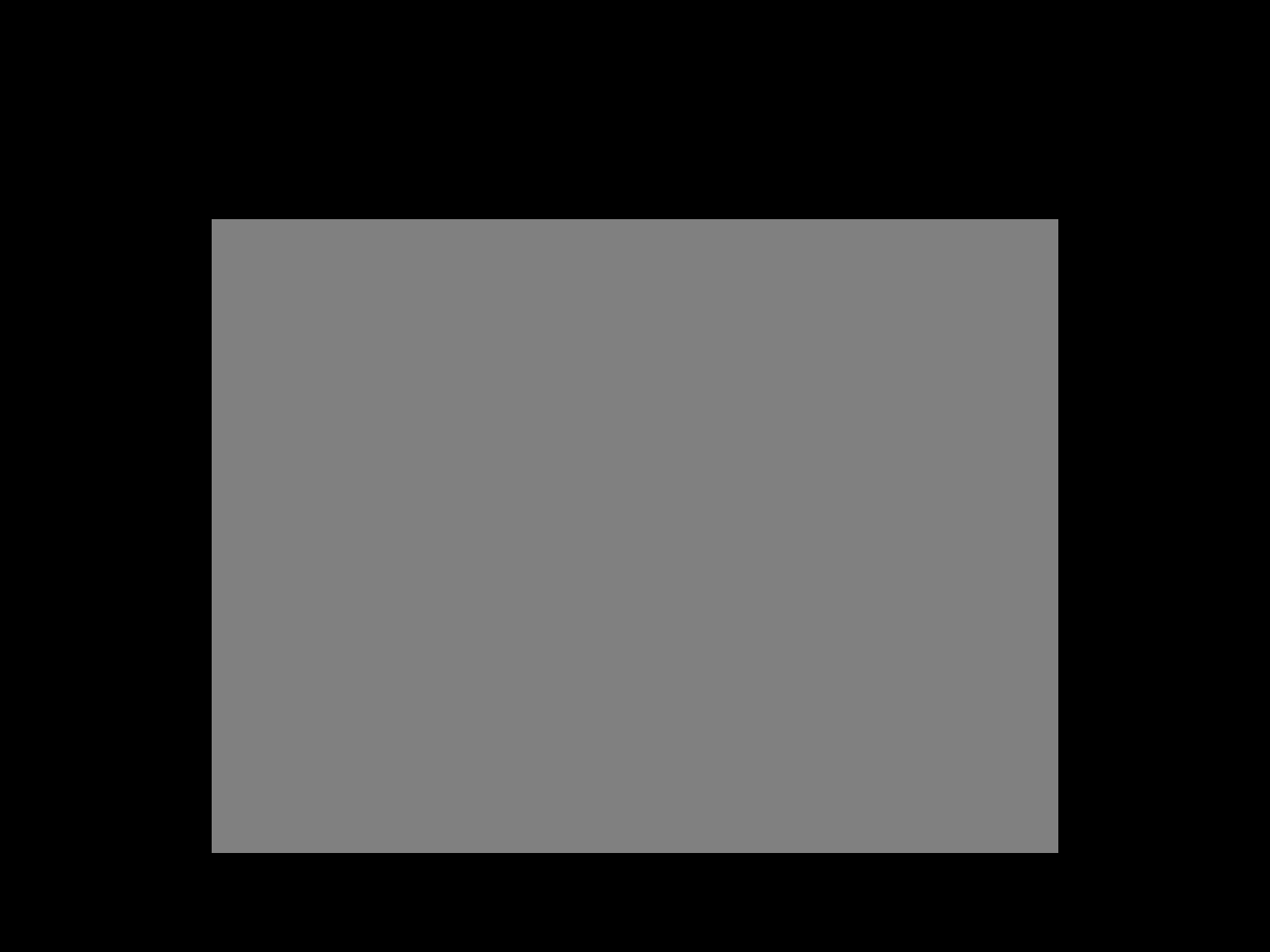

#

## Slide 16
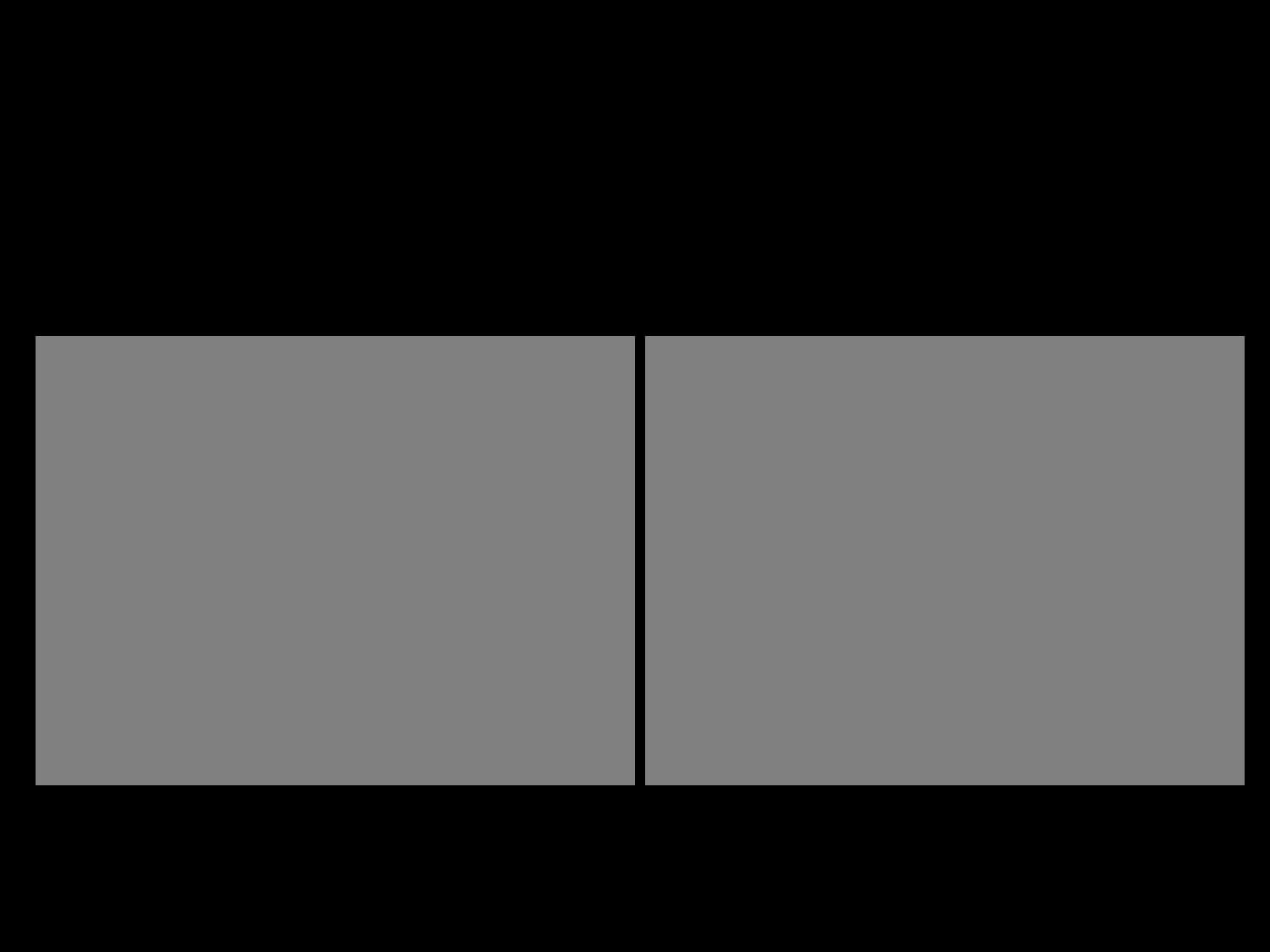

#

## Slide 17
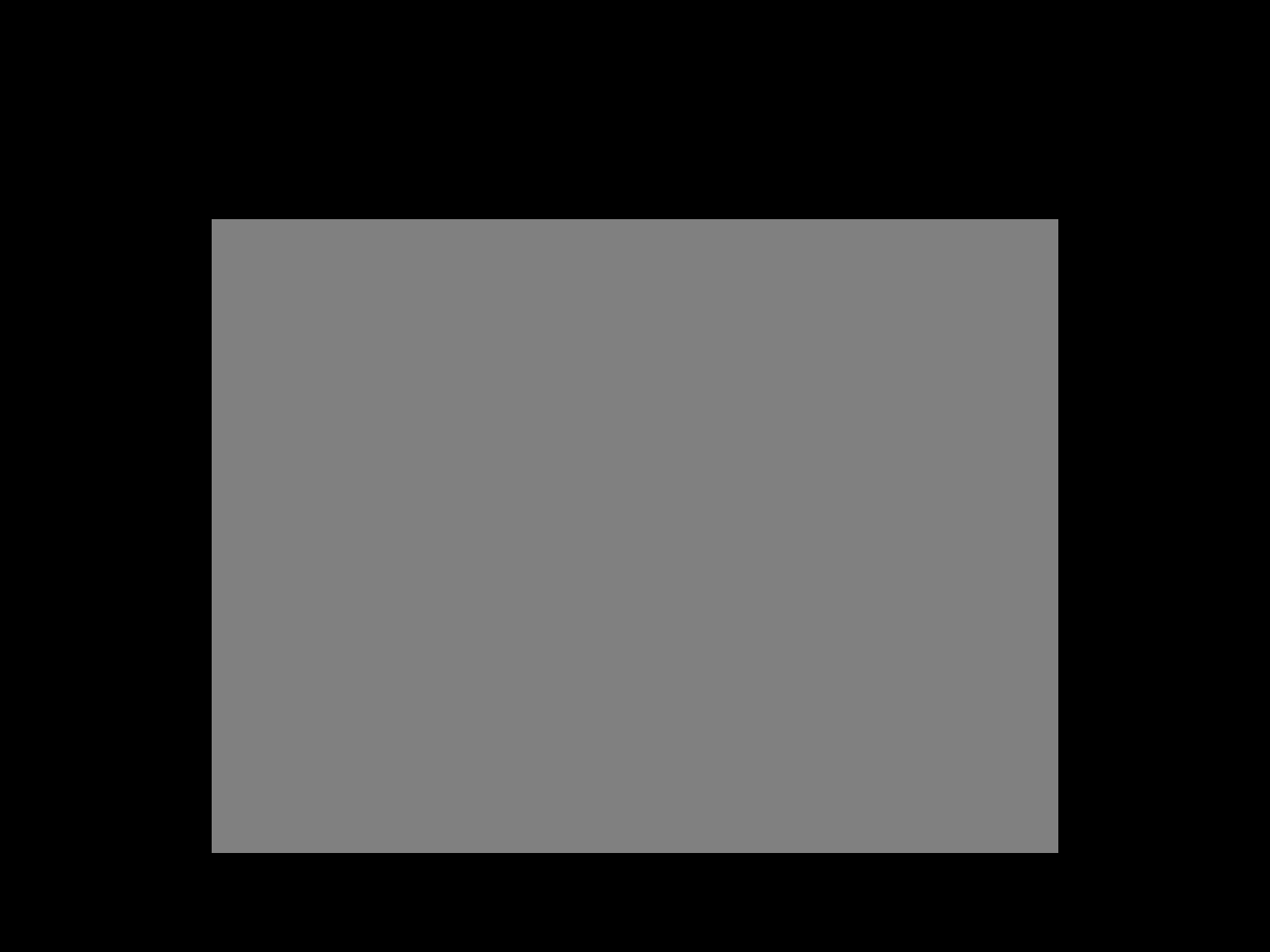

#

## Slide 18
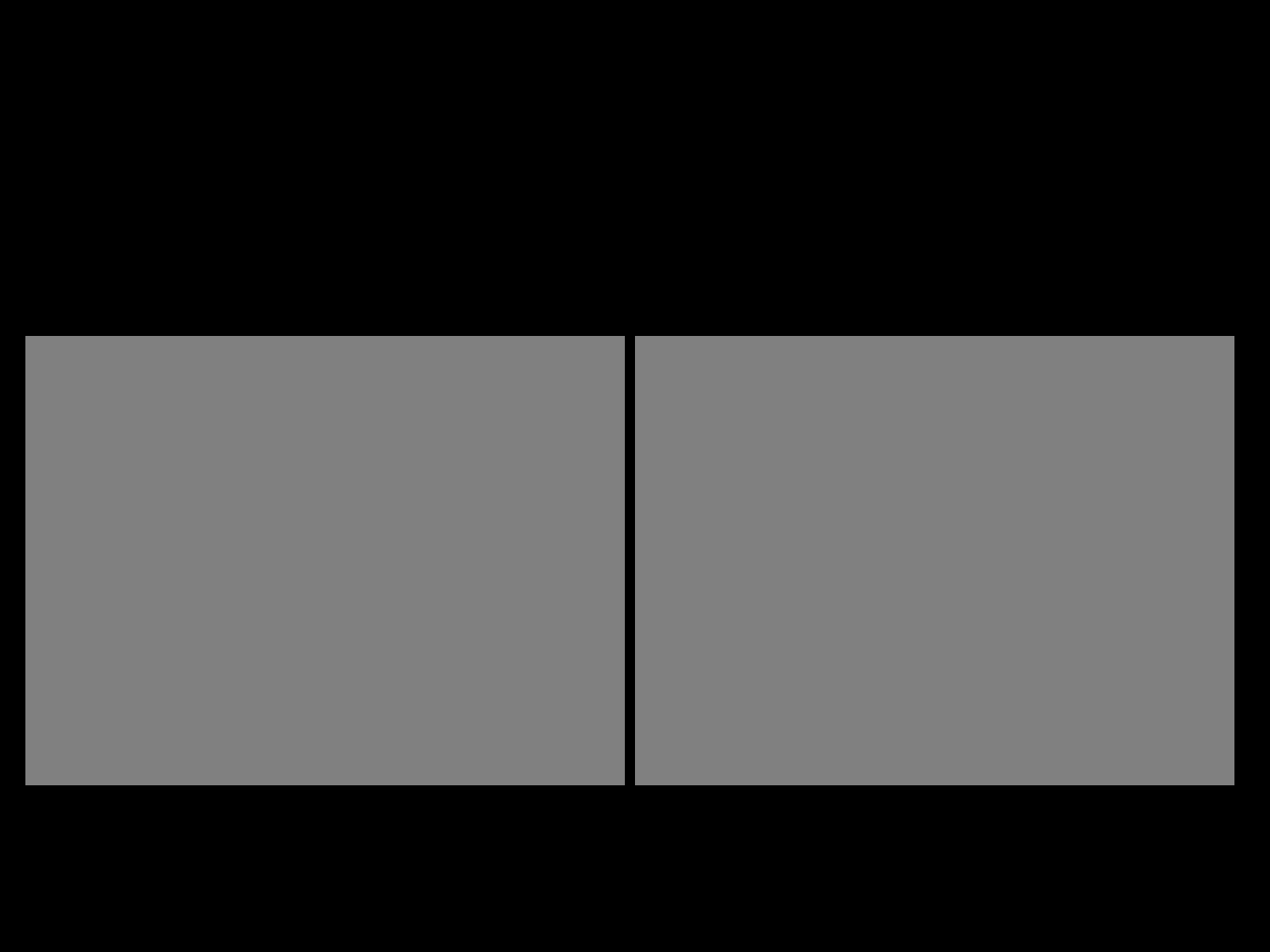

#

## Slide 19
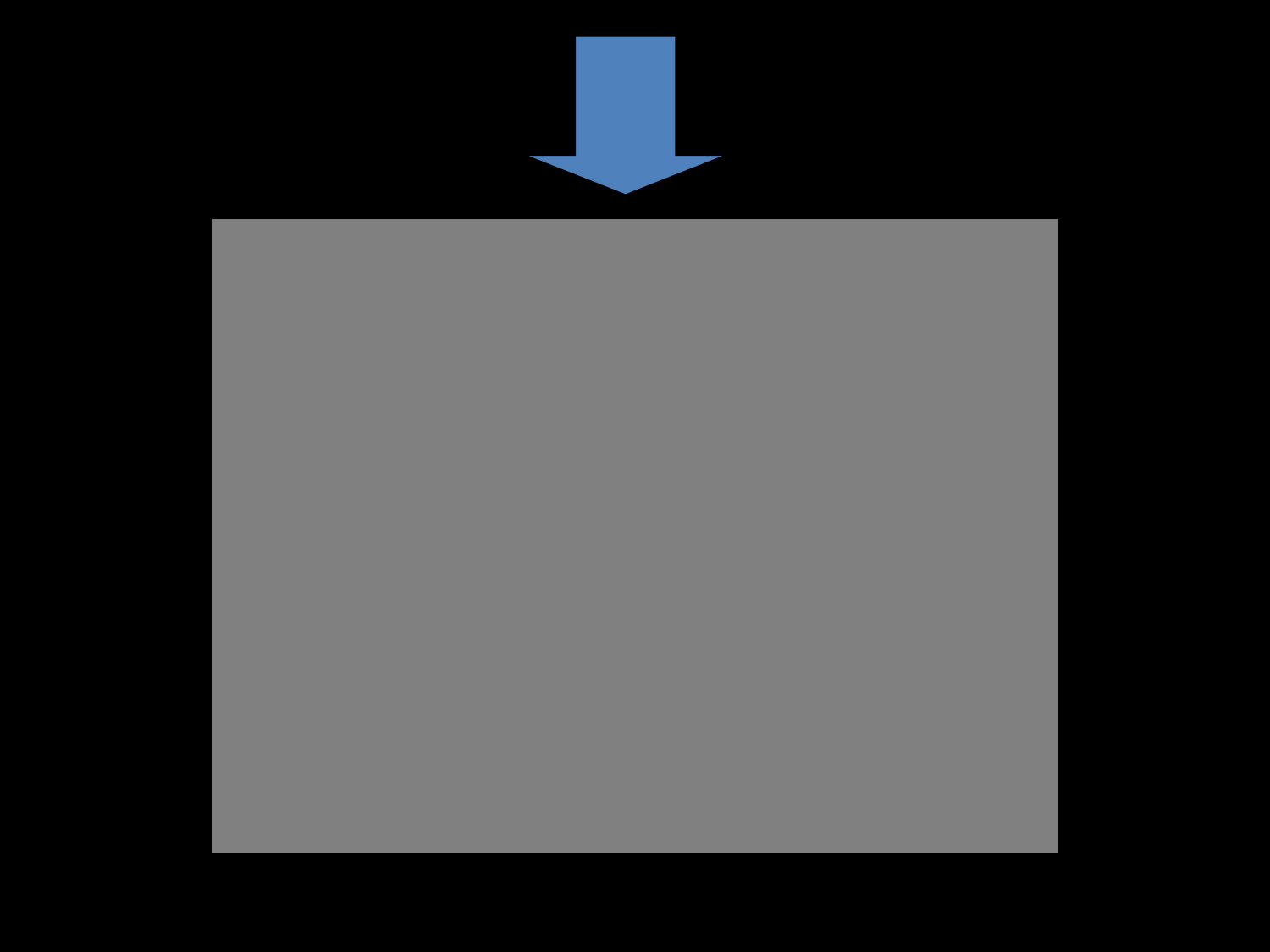

#

## Slide 20
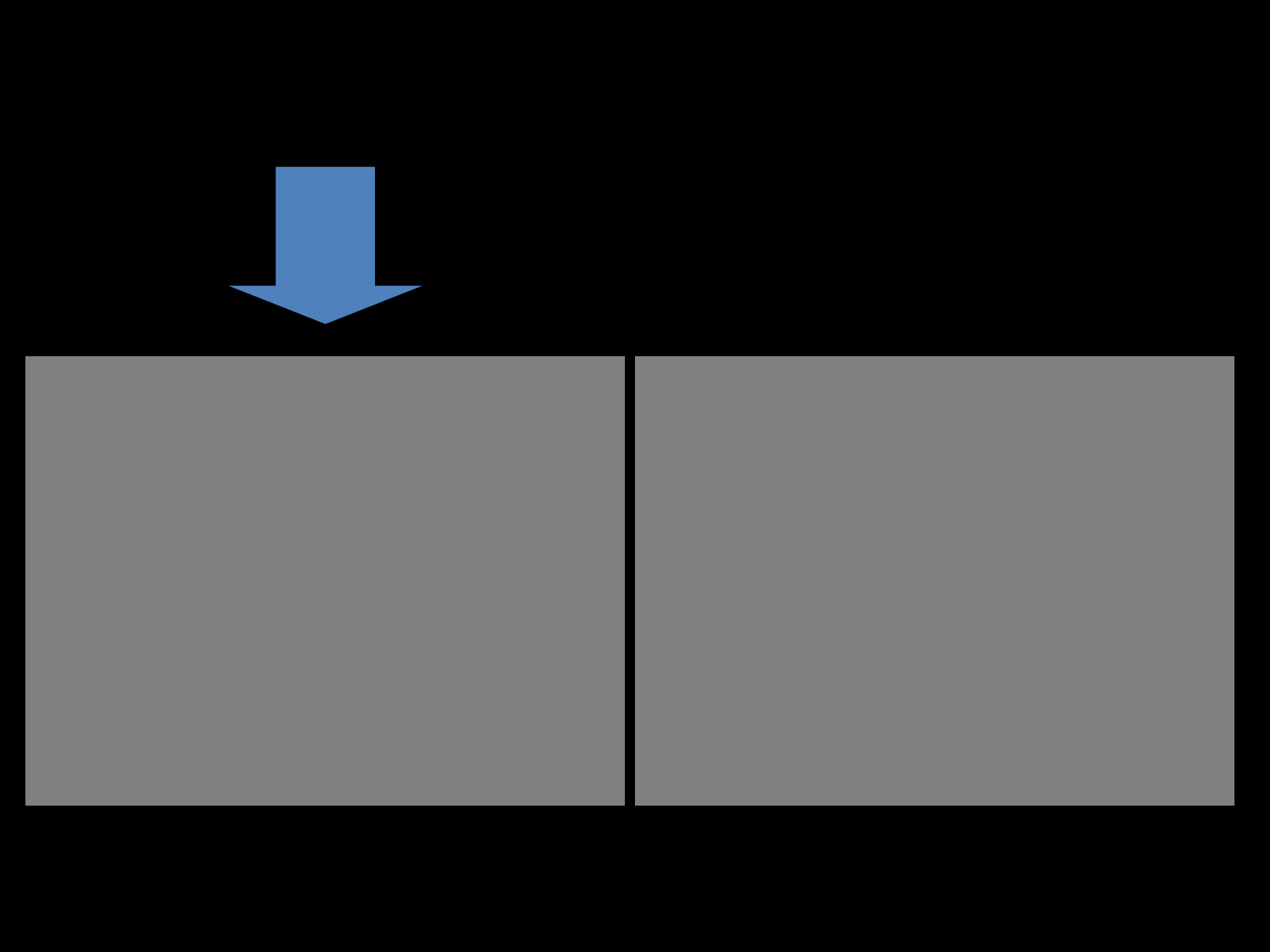

#
